# Age-dependent perturbation of the perceptual and postural vertical by visual roll vection and susceptibility to motion sickness in children

**DOI:** 10.1007/s00415-022-11017-x

**Published:** 2022-02-25

**Authors:** Lutz Schnabel, Max Wuehr, Anna Huppert, Stanislav Bardins, Thomas Brandt, Doreen Huppert

**Affiliations:** German Center for Vertigo and Balance Disorders (DSGZ), Ludwig-Maximilians-Universität, Marchioninistr. 15, 81377 Munich, Germany

**Keywords:** Subjective visual vertical, Roll vection, Postural control, Sensorimotor maturation, Children, Motion sickness

## Abstract

**Background:**

The visual contribution to the perceptual and postural vertical is mediated by a multisensory integration process and may relate to children’s susceptibility to motion sickness that is hypothesized to arise from intersensory conflicts.

**Objective:**

To analyze the maturation of visual contribution to the perceptual and postural vertical in conjunction with the motion sickness susceptibility in childhood.

**Methods:**

In 81 healthy children (aged 2–17 years; 57 females), adjustments of the subjective visual vertical and posturographically tested mediolateral displacements of body sway were measured during free upright stance and large-field visual motion stimulation in the roll plane (roll vection). Motion sickness susceptibility was assessed by taking the history of parents and children.

**Results:**

Vection-induced tilts of the visual vertical showed a linear age-dependent decrease with largest tilts in the youngest (2–7 years; median of 20°) and smallest tilts in the oldest age group (13–17 years; median of 9–10°). Analogously, postural tilts as measured by mediolateral body sway were greatest in the youngest and smallest in the oldest age group. In contrast, motion sickness susceptibility was lowest in the youngest and highest in the oldest age group and exhibited an inverse correlation with vection-induced tilts of the visual vertical.

**Conclusion:**

Roll vection-induced tilts of the visual and postural vertical exhibited a similar age-dependent course with the greatest effects in the youngest and the least effects in the oldest age group, the latter of which exhibited the highest susceptibility to motion sickness.

## Introduction

Large-field visual scenes rotating around the line of sight induce a continuous sensation of self-motion in the roll plane (roll vection) opposite in direction to pattern motion, which results in a “compensatory” tilt of subjective visual vertical (SVV) [1] and postural vertical [2] in direction to pattern motion. Postural destabilization during roll vection has also been demonstrated in children [3]. Other studies found an age-dependent postural destabilization in children experimentally elicited by linear vection [4–8]. Combined measurements of the SVV and postural stability were performed in a small group of healthy children aged 6–8 years compared to adults [9] and in children born prematurely aged 3–4 years compared to an age-matched group born maturely [10]; the children in both studies were more instable than the reference group and showed a higher variability, and lower accuracy in adjusting SVV during visual stimulation in roll. The development of visual contribution to postural stabilization by the perturbating effects of roll vection was tested in 109 healthy children between the ages of 6 months and 18 years. The study revealed three phases: (1) 6- to 12-month-old babies showed none or little disturbance of their newly acquired ability to sit; (2) children between the ages of 2 and 5 years showed maximal dependence of postural stability on vision with a marked ipsilateral deviation or irresistible falls; (3) from 5 to 15 years postural imbalance decreased to their final strength in adulthood with a moderate head and body tilt in response to the rotating stimulus [3]. It was concluded that optokinetically induced vection and its effect on perception and posture depend on the ability of children to freely sit and walk, which is achieved between the ages of 6 and 12 months, and that the age-dependent differences in susceptibility to motion sickness in children are related to this phenomenon.

That children particularly susceptible to seasickness have been already observed in antiquity [11]. Motion sickness is generated either by unfamiliar body motions or by an intersensory mismatch involving conflicting vestibular and visual stimuli [2, 12]. The sensory conflict hypothesis is the most widely accepted theory for the pathogenesis of motion sickness [13]. It was suspected that infants below the ages of 1–2 years are highly resistant to motion sickness because they use the visual system only to a limited extent for dynamic spatial orientation and self-motion perception and thus may be subject to fewer visual-vestibular perceptual conflicts [3]. In two representative cross-sectional population-based surveys on the susceptibility to motion sickness in childhood, the course of motion sickness frequency followed an inverse *U*-shaped curve with a broad resistance in the first year of life, the highest frequency within the range of 4–13 years, and a postpubertal decline [14]. Earlier studies reported a high prevalence of motion sickness in school children between the ages of 7 and 12 years [15] and a gender preponderance of females in school-aged children from 9 to 18 years [16].

In the current study, we tested in participants from early childhood to late adolescence the maturation of the visual contribution to the perceptual vertical (SVV) and postural control (body sway and mediolateral tilt) using a perturbating roll vection stimulus in free upright stance. The major questions were whether deviations of the perceived visual vertical and postural tilts have a similar age-dependent course and whether this is related to susceptibility to motion sickness.

## Methods

### Subjects

Eighty-six healthy children and adolescents aged from 9 months to 17.3 years (59 females) participated in the study. Exclusion criteria were any neurological disorders as assessed by clinical history taking, a relevant visual impairment, or acute vestibular syndromes as assessed by clinical examination. Handedness was not determined since the maturation of handedness can extend up to an age of around 12 years [17].

### Experimental setup and procedures

History of motion sickness was assessed by a custom-made questionnaire which comprised its frequency, triggering factors, symptoms, and age of onset. Frequency of motion sickness in typical triggering situations was categorized as “never”, “once or rarely” and “frequently or always”.

**Fig. 1:**
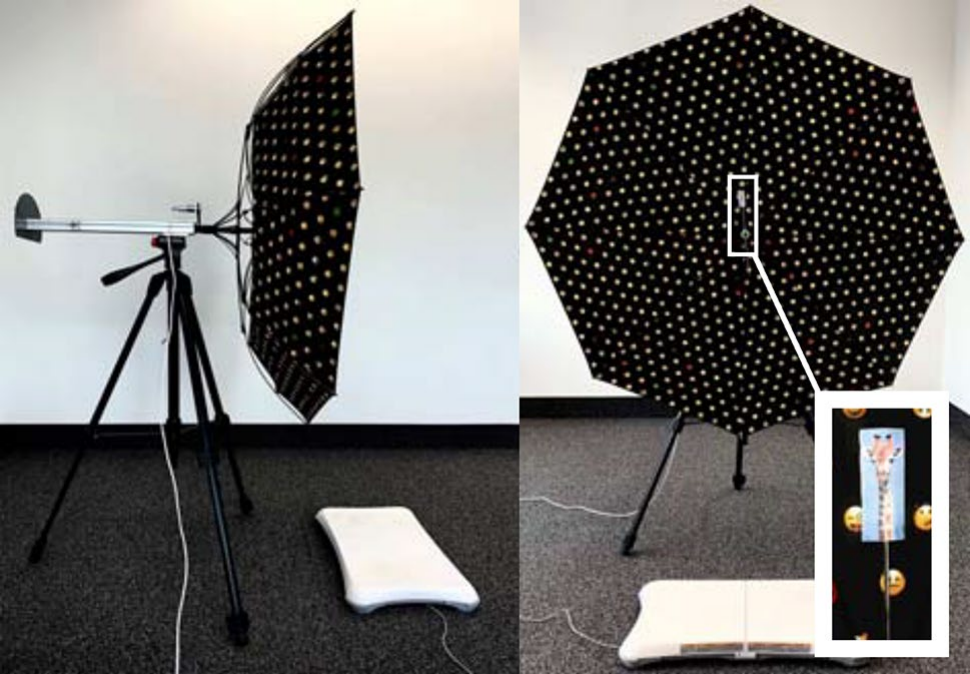
Experimental setup consisting of a height-adjustable inverted umbrella forming a dome around the horizontal axis for visual stimulation in the roll plane, a subjective visual vertical system with a pointer on the rear side, a piece of cardboard on a metallic rod depicting a giraffe in the center of the dome (see insert) and a posturographic platform (bottom)

SVV and postural vertical were measured by a self-built visual rotating dome that consisted of a hand-sewn inverted umbrella (diameter 1.05 m) made of black material, the inner surface of which was covered with a stimulus pattern of contrasting multicolored emojis (mean diameter 0.01 m; distance between emojis 0.035 m) mounted on a height-adjustable tripod (Fig. 1). The dome was either static or rotating in the roll plane (driven by an electromotor) at an angular velocity of 60°/s to create a clockwise (CW) or counterclockwise (CCW) visual stimulus around the subject’s line of sight. The distance between the inner surface of the dome and the subject’s head was chosen so as to be as small as possible with approximately 0.4 m to occupy the full binocular visual field by the stimulus pattern and to occlude any other visual references.

The subject’s perceptual vertical was assessed binocularly while standing upright in a comfortable and stable position with the feet side-by-side. The SVV system was composed of a piece of cardboard on a metallic rod depicting a giraffe in the center of the dome to attract the participant’s attention and a measuring scale with a pointer at the rear side of the dome to read out the tilt of the giraffe (in degrees). The giraffe and the pointer at the rear side were both calibrated perpendicularly to the ground (set as 0°) before measurements. To determine the static SVV, the central piece of cardboard with the giraffe was adjusted to the participant’s eye level. The position of the giraffe was first aligned to true vertical and thereafter slowly rotated by the examiner from 60° CW and CCW tilted positions towards the initial position (three times each, in total six measurements) until the child verbally indicated its subjective verticality to offset influences by the initial rod presentation [18]. The dynamic SSV was determined by an analogous procedure of six adjustments with the dome rotating once in CW and once in CCW direction. The average of the six measurements for the static and for the dynamic SVV during CW and CCW rotations were taken for further analysis. The relative difference between the static SVV and the CW/CCW dynamic SVV was calculated as follows:

$$\mathrm{Relative\,adjustments\,of\,SVV }\left[^\circ \right]=\left|\mathrm{Static\, adjustments\, of\, SVV }\left[^\circ \right]-\mathrm{Dynamic\, adjustments\, of\, SVV }\left[^\circ \right]\right|.$$

In analogy to the SVV measurements, the postural vertical (estimated by the amount of mediolateral body sway) was assessed by means of posturography (Wii Balance Board, Nintendo^®^, Kyoto, Japan, sampling frequency: 60 Hz). Subjects stood quietly without a rotation of the dome to assess the individual body sway level before any visual motion stimulation for a period of 10 s (baseline), after which the dome began to rotate either in CW or CCW direction for a period of 35 s each (stimulation). Changes in body sway were computed by calculating the difference between the average center-of-pressure (CoP) position during baseline and stimulation period in mediolateral dimension. The difference was further divided by the participant’s body height to normalize the sway deviation with respect to different individual body sizes.

### Statistical analysis

Eighty-one of the 86 participants (age between 2.4 and 17.3 years; 57 females) were able to complete the experimental procedures and thus included in the further statistical analysis. Since measures of SVV adjustments and mediolateral body sway deviations did not follow a normal distribution (as determined by the Kolmogorov–Smirnov test), non-parametric tests were used for statistical analysis. Descriptive statistics are reported as medians and total data ranges. To evaluate age-dependent effects in the outcome measures, participants were stratified into three groups that covered comparable age ranges (2–7 years, *n* = 24; 8–12 years, *n* = 29; 13–17 years, *n* = 28). Differences between stratified age groups of the SVV adjustments and the mediolateral body sway deviations were assessed by the Kruskal–Wallis test with a Dunn-Bonferroni post-hoc correction for multiple comparisons. The dependency of both measures on age was further analyzed by regression analysis. Furthermore, potential relations between SVV adjustments, mediolateral body sway deviations, and the frequency of motion sickness were analyzed by Spearman’s rank correlation (*r*_*s*_). Statistical analysis was performed using SPSS (Version 26.0; IBM Corp., Armonk, NY). Results were considered significant at *p* < 0.05 with a small (*r* < 0.1), medium (0.1 ≤ *r* < 0.5) or large (*r* > 0.5) effect size (*r*).

## Results

### Motion sickness susceptibility

**Fig. 2:**
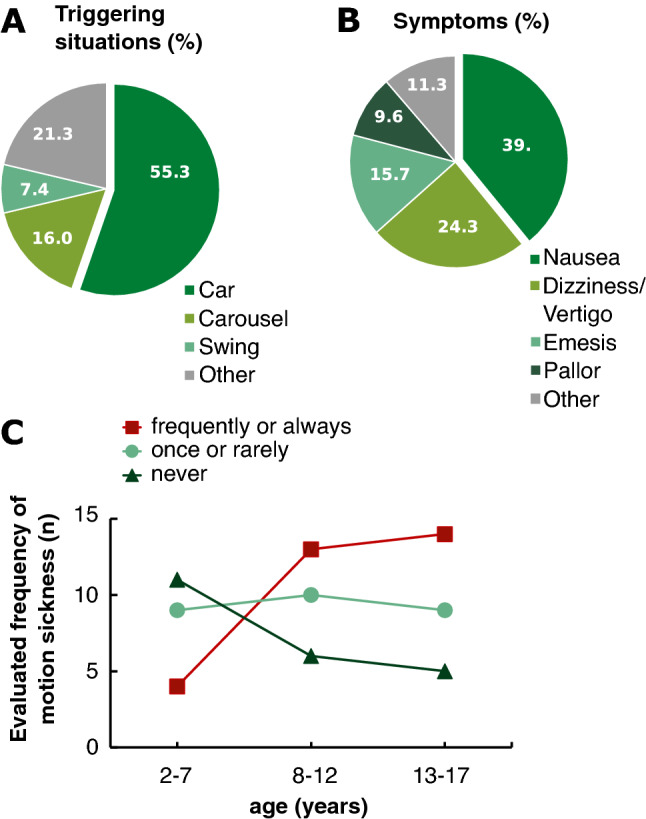
Most stated triggering factors (**A**) and symptoms (**B**) in percent (%) of 59 children and adolescents (3–17 years; 46 females) who experienced motion sickness assessed by a custom-made questionnaire. **C** Frequency of the occurrence of motion sickness in 81 children and adolescents depicted separately as “frequently or always” (red line; *n* = 31; 22 females), “once or rarely” (light green line; *n* = 28; 24 females) and “never” (dark green line; *n* = 22; 11 females) for the three age groups (2–7 years, *n* = 24; 8–12 years, *n* = 29; 13–17 years, *n* = 28) based on the questionnaire. Motion sickness occurred predominantly in the two older age groups but only rarely in the youngest

Among the 81 children and adolescents, 59 participants had experienced motion sickness. The most stated triggering factors for motion sickness were car (55.3%), followed by carousel (16.0%) and swing (7.4%). Presenting symptoms were nausea (39.1%), dizziness/vertigo (24.3%), emesis (15.7%), and pallor (9.6%) (Fig. 2A, B). The frequency of occurrence of motion sickness was stated as follows: 31 participants experienced it “frequently or always” (38.3%; 22 females; age between 3 and 17 years), 28 participants “once or rarely” (34.6%; 24 females; age between 4 and 17 years), and 22 participants “never” (27.2%; 11 females; age between 2 and 15 years). Within the stratified age groups, the motion sickness predominantly occurred in the two older age groups (8–12 years: *n* = 13; 9 females and 13–17 years: *n* = 14; 10 females) and only rarely in the youngest (2–7 years: *n* = 4; 3 females) (Fig. 2C). Of those 59 children who experienced motion sickness, the parents of 46 (37 females) indicated an age of first occurrence of motion sickness that ranged from 1 to a maximum of 13 years with a median of 5 years (25th percentile: 3 years, 75th percentile: 8 years).

### Subjective visual vertical and age

**Fig. 3:**
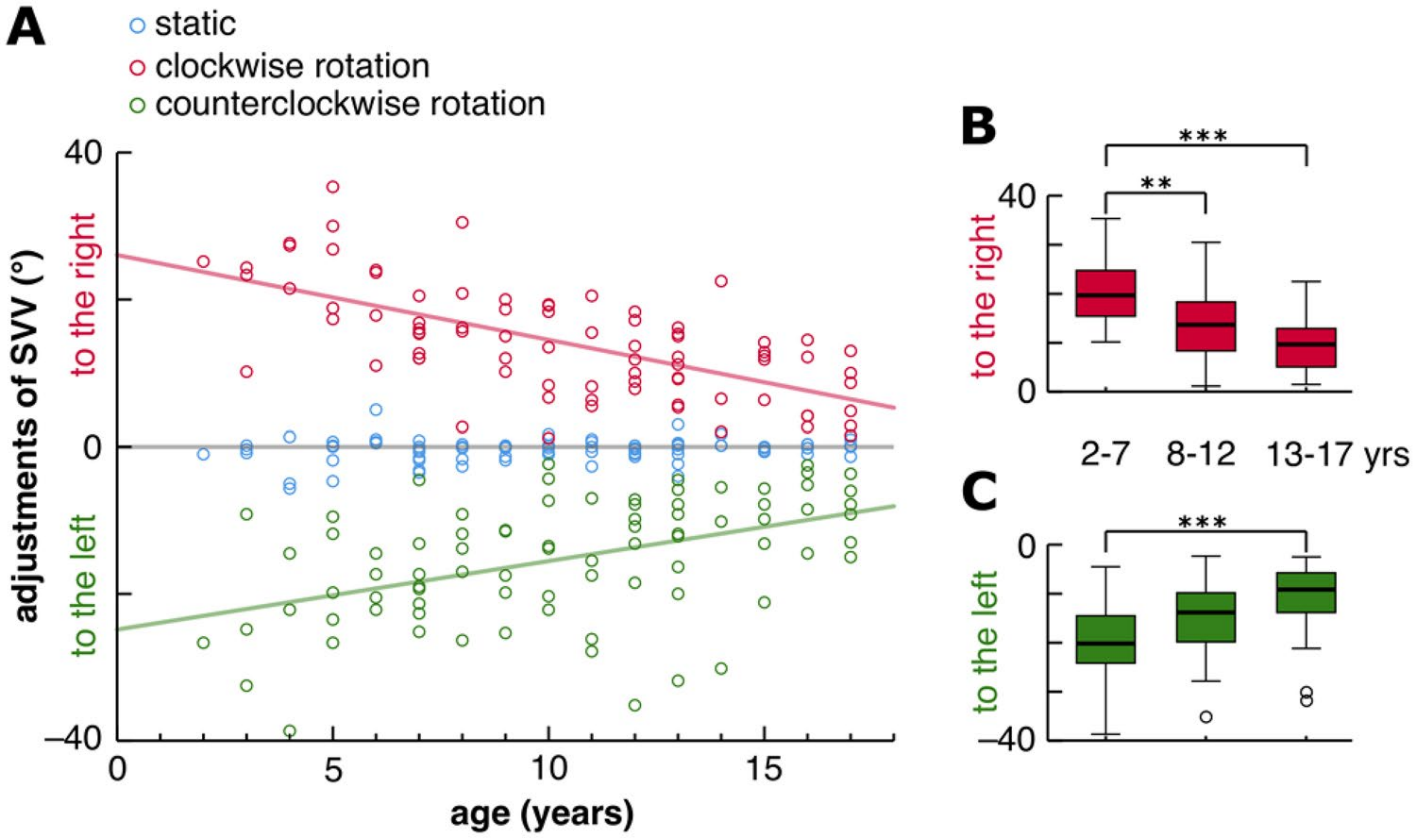
**A** Adjustments of the subjective visual vertical (SVV) of 81 children and adolescents (57 females; 2–17 years) in degrees (°) either to the right or to the left. Each circle represents means of six intraindividual measurements under conditions with static background (blue circles), during roll vection stimulus with clockwise (red circles) and counterclockwise (green circles) rotations of the dome. Trend lines show the fitted linear regression model during clockwise (red line; *R*^2^ = 0.409, *p* < 0.001) and counterclockwise rotations of the dome (green line; *R*^2^ = 0.215, *p* < 0.001). SVV adjustments under static conditions are close to zero for all ages. During roll vection stimulation, SVV adjustments for clockwise and counterclockwise rotations became smaller with increasing age. Age-dependent differences of SVV adjustments stratified by age groups (2–7 years, *n* = 24; 8–12 years, *n* = 29; 13–17 years, *n* = 28) are shown during **B** clockwise rotation and **C** counterclockwise rotation. Significant differences between the age groups are denoted (***p < 0.001; **p < 0.01)

Without any visual stimulation in roll plane, children and adolescents (*n* = 81) exhibited a static SVV with a median of − 0.2 degrees (°) that was close to the true vertical and constant throughout the age groups, even in the youngest group aged from 2 to 7 years (Table 1). During the roll vection stimulus with either CW or CCW rotation of the dome, all participants adjusted SVV during CW rotation to the right and during CCW rotation to the left (Fig. 3A). The absolute dynamic SVV deviations and the relative dynamic SVV deviations (|static – dynamic SVV|, see “Methods”) were comparable (Table 1).

**Table 1:** Static, dynamic and relative adjustments of subjective visual vertical in 81 subjects, grouped by age^a^

Age [years]	Static			Dynamic						Relative adjustments					
				CW			CCW			CW			CCW		
	M	Min	Max	M	Min	Max	M	Min	Max	M	Min	Max	M	Min	Max
2–7 (*n* = 24)	– 0.3	– 5.7	+ 5.0	+ 19.7	+ 10.2	+ 35.3	– 20.2	– 4.5	– 38.7	+ 20.4	+ 10.0	+ 35.3	– 18.5	– 3.8	– 40.0
8–12 (*n* = 29)	– 0.2	– 2.7	+ 1.7	+ 13.7	+ 1.2	+ 30.5	– 13.8	– 2.3	– 35.2	+ 14.5	+ 2.2	+ 30.2	– 12.8	– 1.3	– 35.3
13–17 (*n* = 28)	+ 0.1	– 4.0	+ 3.0	+ 9.7	+ 1.5	+ 22.5	– 9.2	– 2.5	– 31.8	+ 9.9	+ 0.3	+ 22.3	– 9.3	– 2.3	– 30.8
**Total** (*n* = 81)	– 0.2	– 5.7	+ 5.0	+ 13.5	+ 1.2	+ 35.3	– 13.8	– 2.3	– 38.7	+ 14.5	+ 0.3	+ 35.3	– 13.0	– 1.3	– 40.0

*Static* adjustments of the subjective visual vertical under static condition of the dome, *Dynamic* adjustments of the subjective visual vertical with clockwise stimulation and shift to the right or with counterclockwise stimulation and shift to the left, *Relative adjustments* difference between the static and the dynamic SVV; *CW* lockwise, *CCW* counterclockwise, *M* median, *min.* minimum, *max* maximum, + deviation to the right, – deviation to the left

^a^Median, minimum and maximum in degrees of adjustments of the subjective visual vertical are presented

With increasing age, the vection-induced SVV adjustments decreased approximately linearly for dome rotation in both, CW and CCW directions (Fig. 3A). This effect was also apparent when comparing vection-induced SVV adjustments across the three stratified age groups. Accordingly, adjustments decreased from the youngest compared to the oldest age group during CW (*p* < 0.001, *r* = 0.7; Fig. 3B) and CCW stimulation (*p* < 0.001, *r* = 0.6; Fig. 3C) and even between the youngest and the middle age group during CW stimulation (*p* = 0.008, *r* = 0.4; Fig. 3B).

### Postural vertical and age

**Fig. 4:**
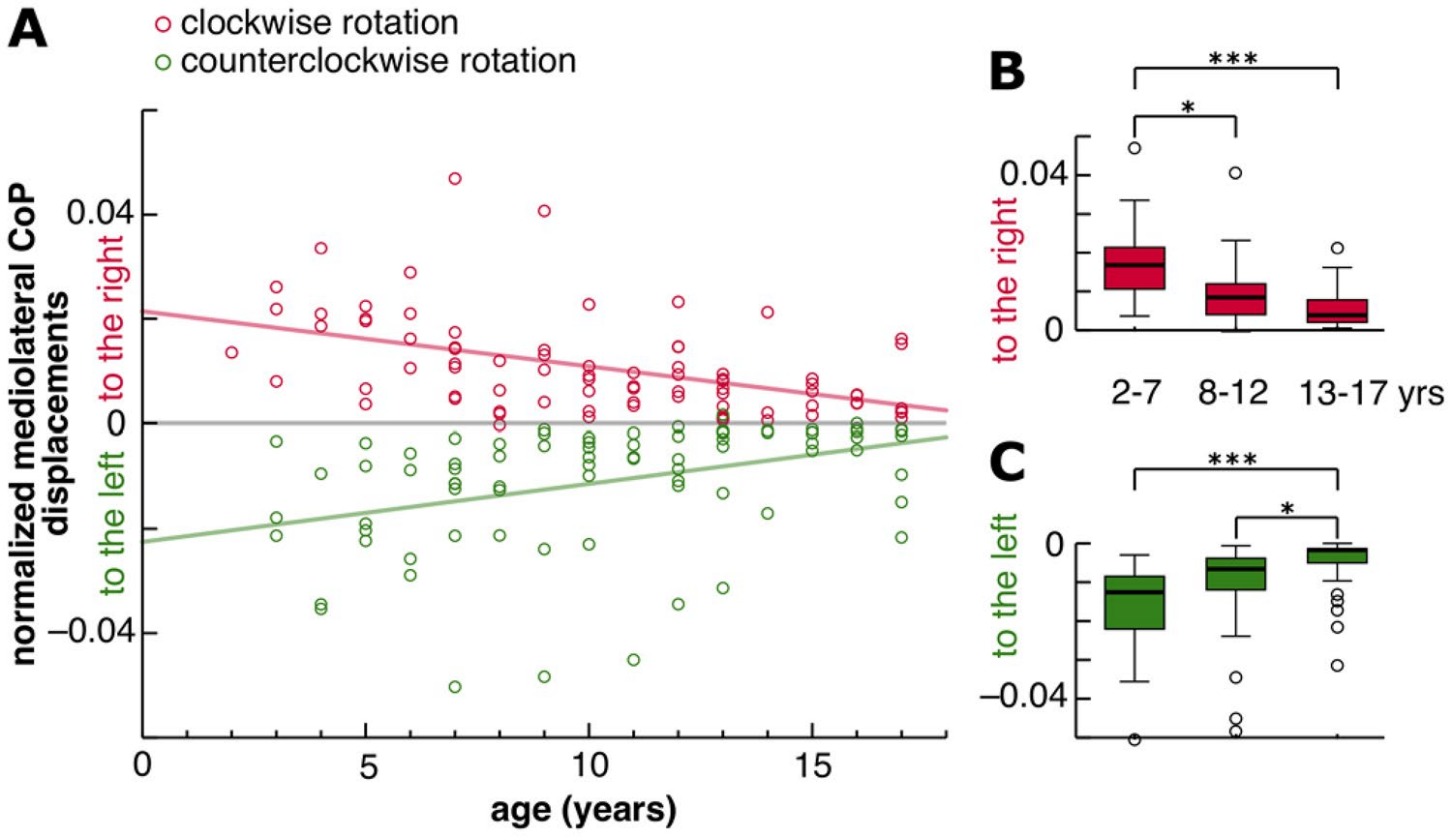
**A** Mediolateral displacements of the center-of-pressure (CoP) either to the right or to the left normalized to body height of 81 children and adolescents (57 females; 2–17 years). Each circle represents the difference in mean mediolateral body sway position during baseline compared to roll vection stimulation with clockwise (red circles) and counterclockwise (green circles) rotations of the dome. Trend lines show the fitted linear regression model during clockwise (red line; *R*^2^ = 0.224, *p* < 0.001) and counterclockwise rotations of the dome (green line; *R*^2^ = 0.146, *p* < 0.001). During roll vection stimulation, body sway deviations became smaller with increasing age. Age-dependent differences of body sway deviations stratified by age groups are shown during **B** clockwise rotation (2–7 years, *n* = 24; 8–12 years, *n* = 29; 13–17 years, *n* = 28) and **C** counterclockwise rotation (3–7 years, *n* = 23; 8–12 years, *n* = 29; 13–17 years, *n* = 28). Significant differences between the age groups are denoted (***p < 0.001; *p < 0.05)

With increasing age, deviations in postural vertical during roll vection measured by the normalized mediolateral displacements of the CoP decreased during CW and CCW rotations of the dome (Fig. 4A). Across the stratified age groups, body sway deviations decreased from the youngest compared to the oldest age group during CW (*p* < 0.001, *r* = 0.7; Fig. 4B) and CCW stimulation (*p* < 0.001, *r* = 0.6; Fig. 4C) and even between the youngest and the middle age groups during CW stimulation (*p* = 0.011; *r* = 0.4; Fig. 4B) and the middle and the oldest age groups during CCW stimulation (*p* = 0.014; *r* = 0.4; Fig. 4C).

### Relationship between roll vection-induced changes in the subjective visual and postural vertical and the frequency of motion sickness

Age-dependent shifts of vection-induced adjustments in SVV and postural vertical were directly correlated with each other for both, CW (*r*_*s*_ = 0.466; *p* < 0.001) and CCW (*r*_*s*_ = 0.339; *p* < 0.001) rotations of the dome. An increased frequency of motion sickness further correlated with decreased vection-induced changes in SVV during CW (*r*_*s*_ = –0.286; *p* < 0.01) and CCW (*r*_*s*_ = –0.230; *p* < 0.05) rotations of the dome. No correlation was found between an increased frequency of motion sickness and vection-induced changes in the postural vertical.

## Discussion

The major findings of this study were that the roll vection-induced deviations of perceptual SVV and body postural vertical showed a similar age-dependent course, being most pronounced in the youngest and least pronounced in the oldest age group. In contrast, the susceptibility to motion sickness recorded by taking the history of the children and their parents was lowest in the youngest age group and increased with age.

### Age-dependent course of SVV tilts during roll vection

All children from the age of 2 years were able to reliably adjust the SVV to the true vertical (total median of – 0.2°; Table 1) at a precision corresponding to that obtained in adults [19]. The global perception of the visual and postural vertical is based on an integrative multimodal graviceptive input from the vestibular (semicircular canals and otoliths), somatosensory, and visual systems by convergence of the modality specific coordinates [20]. When exposed to a rotating dome, children perceived an apparent perception of self-motion in roll in the opposite direction to pattern motion. However, despite the continuous roll-motion of the body, the deviation of the SVV was limited to a total median range of about 14° (Table 1). This limitation can be attributed to the parallel graviceptive feedback about the true earth vertical sensed by the otoliths and somatosensors which counteracts the actual misleading visual information [1]. In the children, the largest SVV tilts were obtained between the ages of 2 and 7 years with a median of 20° (Table 1). The age-dependent adjustments of SVV, which showed a considerable variation, were statistically best explained by a linearly decreasing fit as depicted in Fig. 3 for both CW and CCW rotations. The deviations of the SVV in the oldest group were smaller (median of 9°–10°, Table 1) than those reported earlier in a systematic study on static and dynamic SVV tilts in 110 healthy subjects who exhibited median SVV tilts of 15° [19]. This disparity could be attributed to differences in the experimental settings: subjects tested during free stance in our study and subjects tested when sitting with the head fixed in the earlier study. In free stance, graviceptive input by somatosensors and otoliths might interfere earlier to prevent an impending fall. In a study on a small cohort of children aged 6–8 years, assessment of SVV was also combined with measurements of body sway during roll vection [9]. These authors emphasized a higher variability and a lower accuracy of SVV, and instable postural parameters in the children as compared to young adults.

### Age-dependent course of mediolateral body tilts during roll vection

Postural tilts measured as mediolateral body sway exhibited a similar age-dependent course with the largest tilts in the youngest and the smallest tilts in the oldest age group (Fig. 4). These findings are in agreement with a study of 109 children aged 6 months–18 years during roll vection, in which children aged 2–5 years showed a marked ipsilateral postural deviation or irresistible falls in contrast to 6- to 12-month-old babies while sitting [3]. The former conclusion that the visual loop participates rather late after the multisensory sensorimotor achievement of free upright stance and of locomotion cannot be proved in our study since only children older than 2 years were included. Various studies on visual motion stimulation inducing linear vection also indicated an age-dependent course of postural tilt [4–8, 21, 22].

### Motion sickness and perception of verticality during roll vection

History taking of the children’s parents and the children revealed that the youngest age group from 2 to 7 years had the lowest susceptibility to motion sickness, whereas the two older age groups from 8 years on exhibited a higher susceptibility with a female gender preponderance (Fig. 2C). These data confirm two representative population-based surveys in children (including 7569 and 12,720 households) with the highest frequency of motion sickness occurring between 4 and 13 years and a postpubertal decline of susceptibility [14]. Other surveys on children of 7–12 years also reported a high motion sickness prevalence [15] and a female gender preponderance in a group of children between 9 and 18 years [16]. Thus, children exhibited larger deviations of the perceptual and postural verticals and an increased susceptibility to motion sickness as reported earlier for adults (for reviews see: [14, 19, 20, 23]). There was an inverse correlation between roll vection-induced tilts of the visual but not the postural vertical and the susceptibility to motion sickness. The more a child was susceptible to motion sickness, the less pronounced was the influence of vection on the perceptual vertical. In children susceptible to motion sickness, one might hypothesize that the visual contribution to the perceptual vertical is less reliable than the concurrent integrative graviceptive input from vestibular and somatosensory systems during roll vection. Alternatively, children susceptible to motion sickness might increasingly rely on multisensory information (visual, vestibular, and somatosensory) for self-motion perception and might thus be particularly sensitive to situations of intersensory conflicts. Eventually, the inverse correlation between roll vection-induced tilts of the perceptual vertical and the susceptibility to motion sickness in children might also merely reflect parallel but distinct maturation processes.

## References

[1] Dichgans J, Held R, Young LR, Brandt T (1972). Moving visual scenes influence the apparent direction of gravity. Science.

[2] Dichgans J, Brandt T (1978) Visual-vestibular interaction: effects on self-motion perception and postural control. In: Perception. pp 755–804

[3] Brandt T, Wenzel D, Dichgans J (1976). Visual stabilization of free stance in infants: a sign of maturity (author's transl). Arch Psychiatr Nervenkr (1970).

[4] Lee DN, Aronson E (1974). Visual proprioceptive control of standing in human infants. Percept Psychophys.

[5] Butterworth G, Hicks L (1977). Visual proprioception and postural stability in infancy. A developmental study. Perception.

[6] Stoffregen TA, Schmuckler MA, Gibson EJ (1987). Use of central and peripheral optical flow in stance and locomotion in young walkers. Perception.

[7] Delorme A, Frigon JY, Lagace C (1989). Infants' reactions to visual movement of the environment. Perception.

[8] Baumberger B, Isableu B, Fluckiger M (2004). The visual control of stability in children and adults: postural readjustments in a ground optical flow. Exp Brain Res.

[9] Gaertner C, Bucci MP, Obeid R, Wiener-Vacher S (2013). Subjective visual vertical and postural performance in healthy children. PLoS One.

[10] Bucci MP, Wiener-Vacher S, Trousson C, Baud O, Biran V (2015). Subjective visual vertical and postural capability in children born prematurely. PLoS One.

[11] Brandt T, Bauer M, Benson J, Huppert D (2016). Motion sickness in ancient China. Neurology.

[12] Reason JT (1978). Motion sickness adaptation: a neural mismatch model. J R Soc Med.

[13] Zhang L-L, Wang J-Q, Qi R-R, Pan L-L, Li M, Cai Y-L (2016). Motion sickness: current knowledge and recent advance. CNS Neurosci Ther.

[14] Huppert D, Grill E, Brandt T (2019). Survey of motion sickness susceptibility in children and adolescents aged 3 months to 18 years. J Neurol.

[15] Henriques IF, Douglas de Oliveira DW, Oliveira-Ferreira F, Andrade PMO (2014). Motion sickness prevalence in school children. Eur J Pediatr.

[16] Dobie T, McBride D, Dobie T, May J (2001). The effects of age and sex on susceptibility to motion sickness. Aviat Space Environ Med.

[17] Scharoun SM, Bryden PJ (2014). Hand preference, performance abilities, and hand selection in children. Front Psychol.

[18] Toupet M, Van Nechel C, Grayeli AB (2016). Maturation of subjective visual vertical in children. Otol Neurotol.

[19] Dieterich M, Brandt T (1993). Ocular torsion and tilt of subjective visual vertical are sensitive brainstem signs. Ann Neurol.

[20] Dieterich M, Brandt T (2019). perception of verticality and vestibular disorders of balance and falls. Front Neurol.

[21] Foster EC, Sveistrup H, Woollacott MH (1996). Transitions in visual proprioception: a cross-sectional developmental study of the effect of visual flow on postural control. J Mot Behav.

[22] Higgins CI, Campos JJ, Kermoian R (1996). Effect of self-produced locomotion on infant postural compensation to optic flow. Dev Psychol.

[23] Money KE (1970). Motion sickness. Physiol Rev.

